# Mechanical self-organization of the actin cytoskeleton in artificial cells

**DOI:** 10.2142/biophysico.bppb-v23.0029

**Published:** 2026-09-11

**Authors:** Makito Miyazaki

**Affiliations:** 1 RIKEN Center for Integrative Medical Sciences, Yokohama, Kanagawa 230-0045, Japan; 2 RIKEN Center for Biosystems Dynamics Research, Kobe, Hyogo 650-0047, Japan; 3 Graduate School of Medicine, Science and Technology, Shinshu University, Matsumoto, Nagano 390-8621, Japan

**Keywords:** synthetic biology, symmetry breaking, intracellular positioning, cell migration, *Xenopus laevis*

## Abstract

The actin cytoskeleton generates diverse cellular functions, including intracellular positioning, symmetry breaking, and cell migration, through dynamic self-organization. Despite extensive molecular characterization of actin-binding proteins, the physical principles by which local molecular interactions give rise to cell-scale organization remain incompletely understood. Artificial cells provide a powerful platform for addressing this question because they enable precise control over molecular composition, geometry, and physical boundary conditions while minimizing the complexity inherent to living cells. In this review, we describe how artificial cell systems have been used to investigate the emergence of actin-dependent intracellular organization and motility. Using the cytoplasmic extracts of *Xenopus* eggs encapsulated within cell-sized water-in-oil droplets surrounded by a phospholipid membrane, we reconstructed actomyosin-driven intracellular positioning and spontaneous cell-like migration. By combining quantitative molecular perturbations with theoretical modeling, these studies revealed how competing actomyosin structures, stochastic network formation, and mechanical coupling between the actin cytoskeleton and the membrane collectively determine intracellular organization and directional movement. These findings illustrate how constructive approaches using artificial cells can uncover general physical principles governing the self-organization of cellular architecture and function. This review article is an extended version of the Japanese article, reconstitution of actin cytoskeletal functions in cell-sized confinement, published in SEIBUTSU BUTSURI Vol. 65 p. 192–196 (2025).

## Significance

How cell-scale organization emerges from local molecular interactions remains a central question in biology. This review describes how artificial cells have enabled quantitative dissection of actin cytoskeletal self-organization, revealing simple mechanical principles that govern intracellular positioning and migration. The integration of reconstitution experiments with theoretical modeling provides a powerful framework for understanding the physics of living systems.

## Introduction

Cells are highly organized systems in which hundreds to thousands of molecular species cooperate to build structures spanning several orders of magnitude in length. Although proteins interact only with neighboring molecules through local physical contacts, these interactions collectively give rise to micrometer-scale architectures and coordinated cellular functions. How local molecular interactions generate such large-scale spatial organization remains one of the central questions in cell biology (Figure 1).

The actin cytoskeleton is a prime example of such emergent organization. Dynamic actin networks generate mechanical forces that drive numerous cellular processes, including intracellular transport, positioning of the nucleus and mitotic spindle, cytokinesis, and cell migration. Over the past several decades, remarkable progress has been made in identifying the molecular components that regulate actin cytoskeletal assembly and reorganization [1–8]. However, because these regulatory pathways are extensively interconnected in living cells, it remains challenging to determine which molecular interactions are fundamentally responsible for specific cellular functions. Furthermore, it remains unclear how physical constraints, such as system size and geometry, influence the self-organization of the actin cytoskeleton.

One promising yet challenging strategy for addressing these fundamental questions is the constructive, or bottom-up, approach [9–11]. Cell-sized compartments, such as water-in-oil droplets and giant unilamellar vesicles (GUVs), enable precise control over compartment size, geometry, membrane composition, and protein composition while preserving key physical features of the intracellular environment. By systematically varying biochemical and physical parameters, these reconstituted systems allow direct tests of causal relationships between molecular interactions and emergent functions. Moreover, their close integration with theoretical models provides a rigorous framework for testing physical hypotheses that are often difficult to examine in living cells.

In this review, we summarize our recent studies on actin cytoskeletal self-organization in artificial cells. By encapsulating cytoplasmic extracts from African clawed frog (*Xenopus laevis*) eggs within cell-sized compartments, we successfully reconstituted the intracellular positioning of the nucleus-sized objects and actomyosin-driven migration. These reconstituted systems revealed how dynamic actomyosin networks sense cellular geometry through mechanical interactions and collectively organize intracellular architecture. Together, these findings demonstrate the power of bottom-up reconstitution to uncover the physical principles by which molecular interactions give rise to cellular organization.

## Regulation of intracellular positioning

Spatial organization is a fundamental design principle across all levels of biological organization, from molecular assemblies to whole organisms. At the cellular level, the precise positioning of organelles and macromolecular structures is essential for proper development and function. In mouse oocytes, for example, both the nucleus and the meiotic spindle are positioned with remarkable accuracy (Figure 2), and defects in their positioning can lead to severe developmental abnormalities [12]. Accumulating evidence has established that the actin cytoskeleton is a major determinant of intracellular positioning in mouse oocytes. Nuclear centering has been proposed to result from pressure gradients generated by heterogeneous contraction of the cytoplasmic actomyosin network [13]. In contrast, spindle migration toward the cortex has been attributed either to cytoplasmic streaming driven by actomyosin contractility [14] or to contraction of an actomyosin network connecting the spindle to the cell cortex [15]. Despite these advances, an important conceptual question remains unresolved. How can essentially the same set of cytoskeletal components position intracellular objects either at the cell center or at the edge? Existing models successfully explain individual phenomena but do not provide a unified physical framework that accounts for both types of positioning.

## Size-dependent symmetry breaking in artificial cells

Artificial cells, such as protein-encapsulated cell-sized water-in-oil droplets and GUVs, have emerged as powerful model systems for investigating cytoskeletal self-organization because they allow independent control over molecular composition and physical boundary conditions. Early pioneering studies demonstrated that purified actin or tubulin encapsulated within GUVs can deform membranes through cytoskeletal polymerization [16–18]. Since then, the development of diverse experimental platforms, including GUVs, microfabricated chambers [19], and water-in-oil droplets [20,21], has greatly expanded the range of accessible geometries, boundary conditions, and biochemical environments.

In our studies, we employed water-in-oil droplets encapsulating the cytoplasmic extracts from *Xenopus laevis* eggs as an artificial cell system [22–26]. These extracts faithfully recapitulate many physical and biochemical properties of the intracellular environment while remaining experimentally tractable. Compared with purified reconstitution systems, they retain much of the complexity of the cytoplasm while still allowing quantitative perturbations through the addition of purified proteins, inhibitors, or other defined components.

When the extracts were encapsulated within droplets surrounded by lipid monolayers (Figure 3, A and B) and warmed to induce actin polymerization, an actomyosin network rapidly assembled throughout the droplet and subsequently underwent global contraction [27] (Figure 3C, top). This contraction gathered dispersed organelles into a single dense cluster (Figure 3B). Following the initial contraction, periodic centripetal actin waves emerged from the droplet boundary and propagated toward the center for more than two hours (Figure 3C, bottom and Figure 3D), indicating the establishment of a dynamic steady state in which actin continuously polymerized near the cortex and depolymerized in the interior.

A major advantage of droplet-based artificial cells is that compartment size can be precisely controlled. Surprisingly, the position of the organelle cluster exhibited a striking dependence on droplet diameter. In large droplets, the cluster was consistently localized near the center, whereas in smaller droplets it was localized near the boundary (Figure 4A). The actin waves were still observed in smaller droplets, but their periodicity became less clear and the waves became asymmetric: waves originating from the side closer to the cluster were rapidly absorbed and disappeared upon reaching it, while waves originating from the opposite side propagated across the droplet until they reached the cluster. Here, these clustered organelles resemble simplified intracellular structures such as nuclei or meiotic spindles. Therefore, this system provided an experimental model for investigating the physical principles underlying the symmetry of intracellular positioning. The sharp transition between central and peripheral positioning suggested the existence of two competing mechanisms. We therefore hypothesized that intracellular positioning is determined by a mechanical competition between opposing actomyosin structures rather than by a single positioning mechanism.

## A mechanical tug-of-war between two actomyosin networks

To explain the observed size-dependent transition, we proposed a tug-of-war model consisting of two opposing actomyosin-driven processes [27] (Figure 4B). The first process is driven by periodic centripetal actin waves that originate near the droplet boundary. Molecular perturbation experiments combined with physical modeling suggest that local actin polymerization beneath the membrane, followed by myosin accumulation, initiates waves at the droplet boundary that subsequently propagate toward the center [28]. These waves transport cytoplasmic materials toward the center and therefore promote central positioning of the organelle cluster (Figure 4B, right top). The second process acts in the opposite direction. During peripheral migration, we observed the stochastic formation of actomyosin bridges connecting the organelle cluster to the droplet boundary. Once established, contraction of these bridges generated tensile forces that pulled the cluster toward the cortex (Figure 4B, right bottom). A key insight emerged from considering how these bridges are assembled. Bridge formation requires initially disconnected actin filaments to become mechanically connected through crosslinking proteins over relatively large distances. This process closely resembles a classical percolation problem in statistical physics, in which a system undergoes a transition from disconnected to connected states as network connectivity increases [29]. Within this framework, the characteristic time required for bridge formation (*τ*) is predicted to increase exponentially with the distance between the cluster and the droplet boundary, which scales with droplet size (*D*) (Figure 4C, red line). In contrast, the period of the centripetal actin waves (*T*) increases only weakly with droplet diameter (Figure 4C, blue line). Consequently, intracellular positioning is determined by whichever process occurs first. If bridge formation precedes the arrival of an actin wave, contractile bridges pull the cluster toward the boundary. Conversely, if an actin wave reaches the cluster before bridge formation is completed, the propagating wave sweeps away cytoplasmic actin filaments, effectively resetting bridge formation and promoting central positioning.

This simple kinetic competition naturally predicts the existence of a critical droplet diameter D* at which the dominant positioning mechanism switches. Above this threshold, actin waves dominate, and clusters localize centrally, whereas below the threshold bridge-mediated contraction becomes sufficiently rapid to drive peripheral positioning. To test this prediction experimentally, we altered actin filament length by adding purified regulatory proteins to the extracts (Figure 4D). Formin, which promotes formation of long, unbranched actin filaments, was expected to accelerate bridge formation by increasing network connectivity, whereas gelsolin, which severs actin filaments, should have the opposite effect. As predicted, formin shifted the critical transition toward larger droplets, whereas gelsolin shifted it toward smaller droplets. Importantly, neither perturbation substantially altered the period of the actin waves, indicating that these proteins selectively affected bridge formation. The experimentally observed transition diameters quantitatively agreed with theoretical predictions, providing strong support for the tug-of-war model. Note that, owing to the point symmetry of the system, the cluster position was randomly determined. Our model indicates that small deviations of the cluster from the droplet center, as well as local differences in actin filament density or length, can provide a local bias that determines the direction of network percolation.

Interestingly, the bridge-mediated positioning mechanism closely resembles a previously proposed model for meiotic spindle migration in mouse oocytes, in which formin-generated actin networks mechanically connect the spindle to the cortex and contract to pull the spindle toward the cell periphery [15]. Our results provide a quantitative physical interpretation of this process based on percolation-mediated network formation, in which a combination of local crosslinking events between neighboring actin filaments drives a transition from a disconnected to connected network capable of transmitting forces over long distances. This mechanism further predicts that local activation of formin can bias the direction of spindle movement by promoting network percolation. In contrast, the contribution of periodic actin waves to intracellular positioning has not yet been demonstrated in living cells, suggesting an intriguing direction for future investigation.

## Symmetry breaking drives cell-like migration

The tug-of-war model predicts that the interaction between the actomyosin bridge and the membrane is another key determinant of intracellular symmetry breaking. To test this prediction, we increased actin–membrane coupling by incorporating phosphatidylinositol 4,5-bisphosphate (PI(4,5)P_2_) into the lipid membrane. PI(4,5)P_2_ is known to recruit multiple actin-binding proteins, including WASP family proteins, which activate Arp2/3-mediated actin polymerization [30], and α-actinin, an actin crosslinking protein [31], thereby strengthening the mechanical linkage between the actin cytoskeleton and the plasma membrane. In the presence of PI(4,5)P_2_, periodic actin waves were still observed. These waves likely arise through a similar mechanism to that observed in the absence of PI(4,5)P_2_, in which local actin polymerization beneath the membrane promoted by PI(4,5)P_2_, is followed by myosin accumulation to initiate wave formation. As predicted by the tug-of-war model, enhanced membrane coupling promoted peripheral positioning of the organelle cluster even in large droplets. Unexpectedly, however, the addition of PI(4,5)P_2_ to the membrane produced a more striking phenotype. Rather than remaining stationary, the droplets spontaneously migrated through the gap between two glass surfaces, exhibiting cell-like locomotion [32].

## Reconstituting adhesion-independent cell migration

Animal cell migration can be broadly classified into two major modes. The first is driven by flat membrane protrusions known as lamellipodia, in which actin polymerization pushes the plasma membrane forward to generate protrusive forces (Figure 5, left). The second is driven by the actomyosin cortex, a contractile meshwork located beneath the plasma membrane, in which actomyosin contractility provides the driving force for migration (Figure 5, center). For many years, it was widely believed that both migration modes require the formation of focal adhesions to transmit propulsive forces to the extracellular environment. However, recent studies have identified a cortex-driven migration mode that does not require focal adhesions [33–40] (Figure 5, right). This adhesion-independent, fundamentally different migration mode has been shown to be used by primordial germ cells and immune cells during migration through tissues, and accumulating evidence suggests that it is also exploited by cancer cells during invasion and metastasis. Owing to its important roles in development and disease, this mode of migration has attracted considerable attention.

In this migration mode, propulsion is thought to be transmitted to the surrounding environment through physical friction between the cell surface and its surroundings. However, the mechanism by which actomyosin contractility is converted into whole-cell translational motion remains poorly understood. Because this migration strategy does not require the assembly of the highly complex focal adhesion machinery, it has been proposed to represent one of the simplest and possibly most ancient forms of eukaryotic cell migration [41]. Our spontaneously migrating artificial cells provide a minimal experimental system for investigating this long-standing question by isolating the essential physical processes underlying the conversion of isotropic actomyosin contraction into persistent translational motion (Figure 6A).

## From contractility to directional motion

Detailed imaging revealed that peripheral positioning of the organelle cluster fundamentally altered actin-wave dynamics. In droplets with centrally positioned clusters, centripetal actin waves propagated symmetrically from all directions. In contrast, once the cluster moved toward the boundary, waves approaching from the cluster side were rapidly absorbed and extinguished, whereas those originating from the opposite side continued to propagate. Consequently, the initially symmetric oscillatory pattern spontaneously transitioned into a persistent unidirectional actin flow (Figure 6, B, C, and D). Remarkably, the droplets migrated in the direction opposite to actin-wave propagation. This behavior closely resembles the retrograde actin flow observed in migrating cells, in which cortical actin continuously flows from the leading edge toward the rear while the cell moves forward [38,40]. Partial inhibition of either actin polymerization or myosin activity using inhibitors demonstrated that migration depended on actomyosin contractility. Moreover, droplet velocity exhibited a strong positive correlation with actin-wave velocity, suggesting that propagating contractile waves generate the driving force for locomotion.

To determine how contractile forces were transmitted to the external environment, fluorescent beads were introduced into the narrow gap between the droplet and the glass substrate. When PI(4,5)P_2_ was present, the beads moved together with the rearward-propagating actin waves, indicating that actomyosin-generated forces were transmitted from the cytoskeleton to the membrane and subsequently to the substrate. In contrast, droplets lacking PI(4,5)P_2_ still generated actin waves but neither displaced the beads nor exhibited directional movement of the droplet. These observations demonstrate that actomyosin contractility alone is insufficient to drive migration. Persistent locomotion requires efficient mechanical coupling between the actin network and the membrane, allowing intracellular contractile forces to be transmitted to the external environment.

## Integrating theory and molecular perturbation

To quantitatively describe the migration mechanism, we developed a hydrodynamic model based on force balance [32]. The migration velocity of an artificial cell is determined by the balance between two opposing forces: the propulsive force generated at the cell-substrate interface and the viscous drag exerted by the surrounding oil phase. Solving the governing hydrodynamic equations yielded an analytical relationship between migration velocity and experimentally controllable parameters, including solvent viscosity and geometric confinement (Figure 7A). Among the model parameters, all except a single coefficient (*α*) could be measured independently. The parameter *α* represents the effective friction coefficient between the actin network and the substrate, mediated by the lipid monolayer. More specifically, *α* encompasses both specific and non-specific interactions between the actin network and the hydrophilic phospholipid headgroups, and non-specific interactions between the hydrophobic phospholipid tails and the PDMS surface. If the proposed mechanism is correct, data obtained under different confinement geometries and solvent viscosities should all be described by a single value of *α*, because *α* is independent of the confinement geometry and depends solely on chemical parameters, such as actomyosin activities and lipid composition. This prediction was borne out experimentally [32]. Migration velocities measured under a wide range of physical conditions collapsed onto the theoretical curve using the same value of α (Figure 7B), providing strong quantitative support for the model.

The model further predicts that directional migration does not depend on the molecular identity of membrane-associated proteins, but rather on the existence of a mechanical linkage between the actin network and the membrane. To test this prediction, we replaced PI(4,5)P_2_-mediated interactions with an entirely synthetic coupling system based on Ni-NTA lipids embedded in the membrane and His-tagged actin-binding proteins introduced into the extract. Both His-tagged WASP and His-tagged α-actinin successfully restored symmetry breaking, unidirectional actin flow, and droplet migration [27,32], demonstrating that directional migration depends on mechanical coupling rather than the specific biochemical pathways or molecular components that mediate it. Mechanical interactions alone are sufficient to establish stable cellular polarity and drive directional migration in this minimal reconstituted system.

## Mechanical interactions enable spatial organization in artificial cells

The studies described in this review illustrate how bottom-up reconstitution, quantitative molecular perturbation, and theoretical modeling can be integrated to uncover the physical principles governing cytoskeletal self-organization. In the first part, we showed that intracellular positioning emerges from the competition between two dynamically assembled actomyosin structures. Centripetal periodic actin waves transport material toward the center, whereas stochastic actomyosin bridges generate contractile forces that pull intracellular objects toward the boundary. The balance between these two processes, determined by their characteristic timescales, gives rise to a size-dependent transition in intracellular positioning. This framework provides a unified physical explanation for how the same cytoskeletal machinery can generate either central or peripheral localization.

In the second part, we demonstrated that strengthening actin–membrane coupling converts intracellular symmetry breaking into whole-cell polarity and directional migration. Enhanced mechanical coupling enables contractile forces generated by the actomyosin network to be transmitted through the membrane to the external environment, thereby converting intracellular contractility into translational motion. Importantly, this transition depends not on the biochemical identity of the linker molecules, but on the strength of mechanical coupling between the cytoskeleton and the membrane.

Taken together, these findings highlight a central conceptual principle: the actin cytoskeleton functions not only as a force-generating machinery but also as a mechanical information-processing system. Through continuous assembly, disassembly, and force transmission, dynamic actomyosin networks sense the size, geometry, and boundary conditions of the cellular environment and self-organize intracellular architecture accordingly. In this view, cell-scale organization emerges not through centralized control but from collective mechanical interactions among numerous cytoskeletal components. This perspective shifts the emphasis from identifying individual regulatory molecules toward understanding how dynamic mechanical interactions among many molecules collectively generate cellular architecture and function.

## Concluding remarks and future perspectives

Reconstitution approaches have become an increasingly powerful strategy for investigating complex biological systems. Rather than simplifying biology by removing components, constructive reconstitution asks a fundamentally different question: what is the minimal set of molecular interactions required to generate a given biological function? It seeks to determine how physical constraints regulate biological phenomena and to what extent they shape cellular behavior. This perspective complements traditional perturbation-based cell biology and provides a direct route toward identifying the causal mechanisms underlying emergent cellular behaviors.

Artificial cells are particularly well suited for this purpose because molecular composition, compartment geometry, membrane properties, and mechanical boundary conditions can all be independently controlled. Such precise experimental control enables rigorous testing of theoretical predictions that are often difficult to examine in living cells. The close integration of quantitative experiments with physical modeling therefore allows mechanistic hypotheses to be evaluated at an unprecedented level. More broadly, artificial cells provide a physical counterpart to computational simulations. Whereas computational simulations describe molecular interactions mathematically, artificial cells reconstruct those interactions directly in real space using the molecules themselves. This unique feature enables biological functions to emerge naturally from molecular interactions while maintaining experimental accessibility and quantitative control.

The principles discussed in this review are unlikely to be limited to intracellular positioning and migration. Similar physical principles may underlie diverse morphogenetic processes, including cytokinesis, tissue morphogenesis, wound healing, and collective cell migration. As reconstitution techniques continue to advance, artificial cells will increasingly serve as experimental platforms for dissecting these complex phenomena and for establishing general physical principles of cellular self-organization. Ultimately, understanding how molecular interactions spontaneously generate cellular architecture remains one of the central challenges in biology. By reconstructing life-like behaviors from defined molecular components, artificial cells provide a unique experimental framework for uncovering the physical principles that connect molecular interactions to cell-scale organization, bringing us closer to understanding how living systems emerge from collections of interacting molecules.

## Conflict of interest

The authors declare no competing financial interests.

## Author contributions

M.M. conceptualized the topic of this article and wrote the manuscript.

## Data availability

The data generated or analyzed during the current study are available from the corresponding author upon reasonable request.

## Figures and Tables

**Figure 1:**
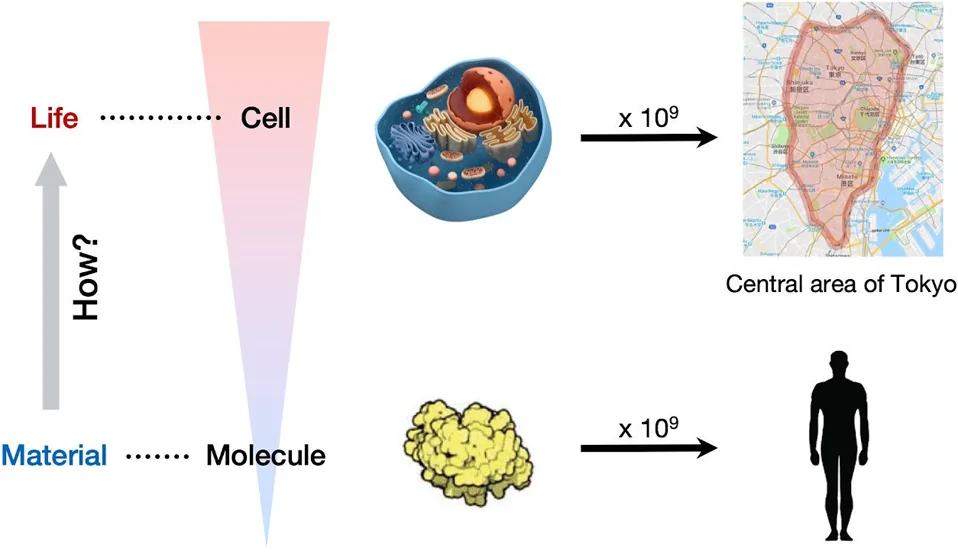
Comparison of protein and cell sizes. If a protein were scaled up to the size of a human, a cell would be comparable in size to a megacity. In such a world, it is far from obvious how these tiny molecules coordinate their activities to organize cell-scale ordered structures and generate biological functions.

**Figure 2:**
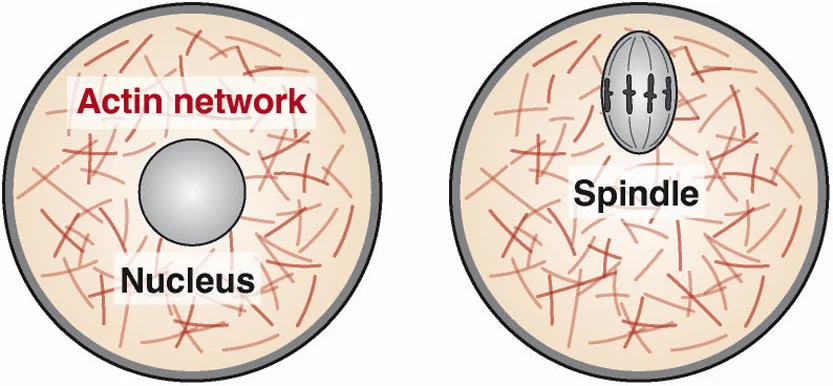
Positioning of intracellular structures in mouse oocytes. The actin cytoskeleton positions the nucleus at the cell center and the meiotic spindle near the cell periphery.

**Figure 3:**
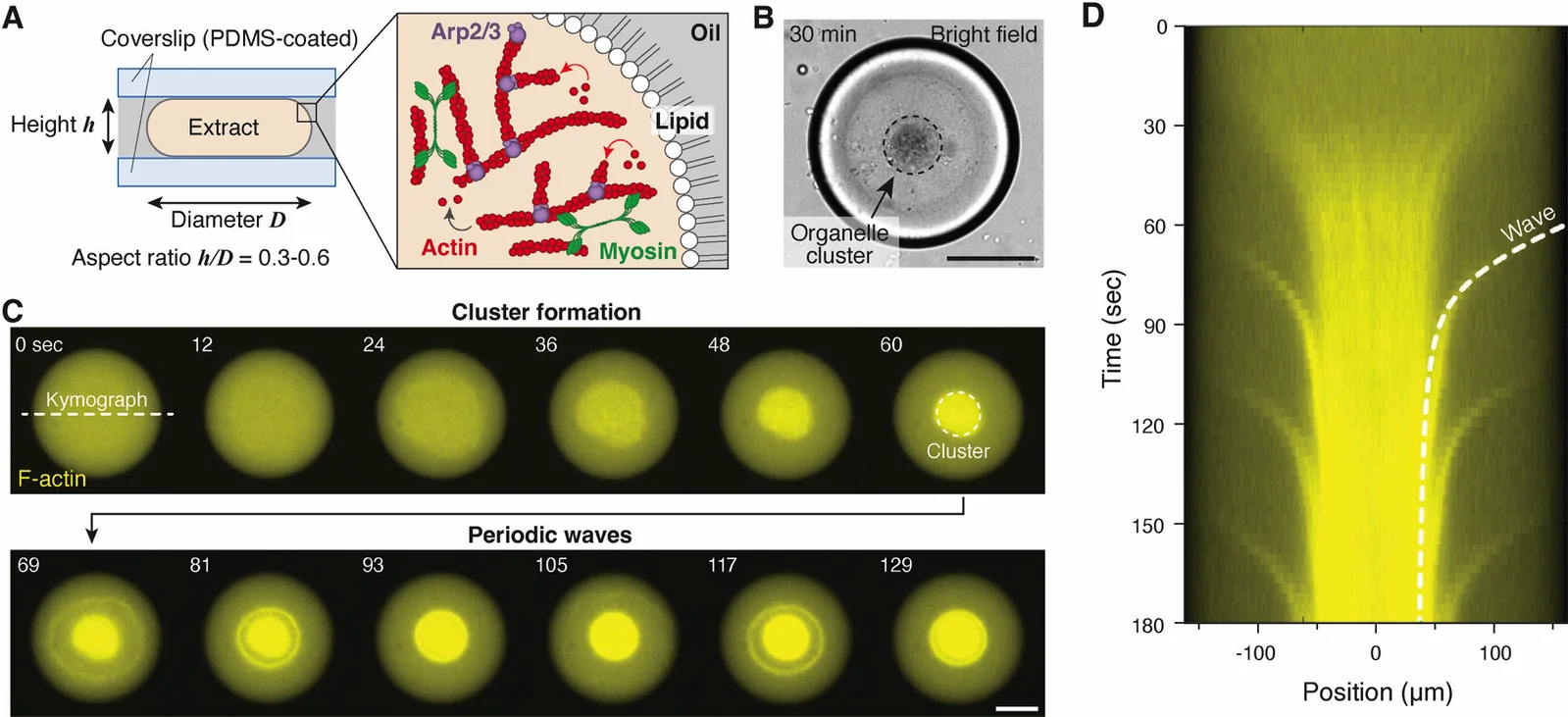
Water-in-oil droplets encapsulating cytoplasmic extracts. (A) Schematic illustration of the experimental system. To isolate actin-dependent organization, microtubule polymerization was pharmacologically suppressed, and an ATP regeneration system was included to sustain actin dynamics for more than two hours. To facilitate quantitative analysis, droplets were compressed between two coverslips to form disk-shaped compartments that could be approximated as a quasi-two-dimensional system. (B) Bright-field image. (C, D) Time-lapse fluorescence images (C) and the kymograph (D) showing the distribution of actin filaments visualized with TMR-Lifeact, a short F-actin binding peptide fused with a fluorescent dye. Scale bars, 100 μm.

**Figure 4:**
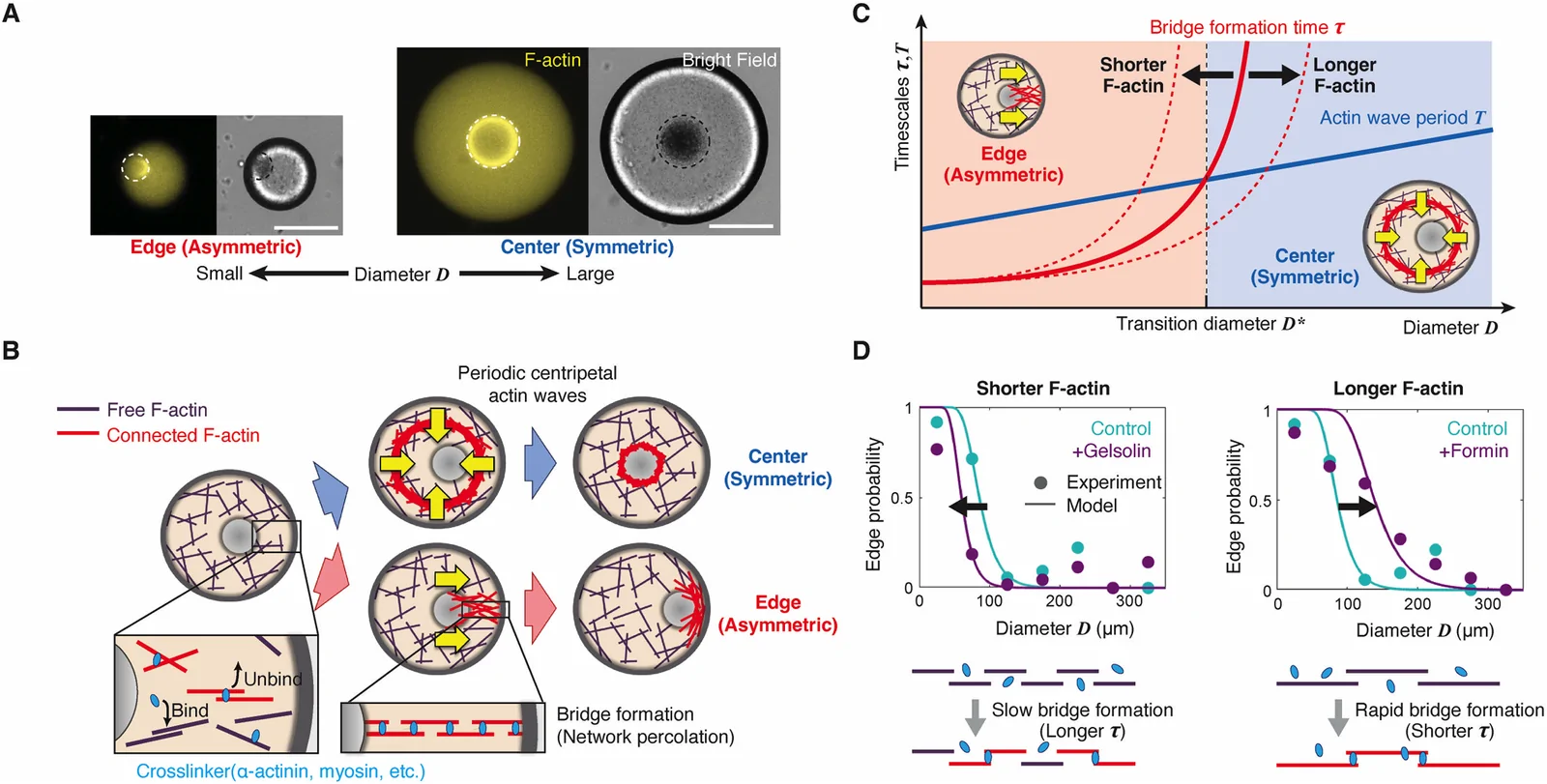
Positioning mechanism based on a “tug-of-war” between two distinct actin networks. (A) Size-dependent symmetry breaking of the cluster positioning. (B) Tug-of-war model. Right top: Periodic actin waves generated at the droplet periphery propagate toward the center, transporting the cluster inward. Right bottom: Sparsely distributed cytoplasmic actin filaments become stochastically connected by cross-linking proteins, leading to the formation of a bridge between the cluster and the droplet boundary that pulls the cluster toward the periphery. If an actin wave is generated before the bridge is completed, the wave incorporates and clears the cytoplasmic actin filaments as it propagates, resetting bridge formation to the initial state. (C) Mechanism determining the transition point in the tug-of-war model. The cluster positioning is determined by whichever characteristic timescale is shorter: actin wave period (*T*) or bridge formation time (*τ*). (D) Experimental validation of the model by molecular perturbation. The transition point was shifted by altering F-actin length using regulatory proteins. The experimental results agreed well with the mathematical model.

**Figure 5:**
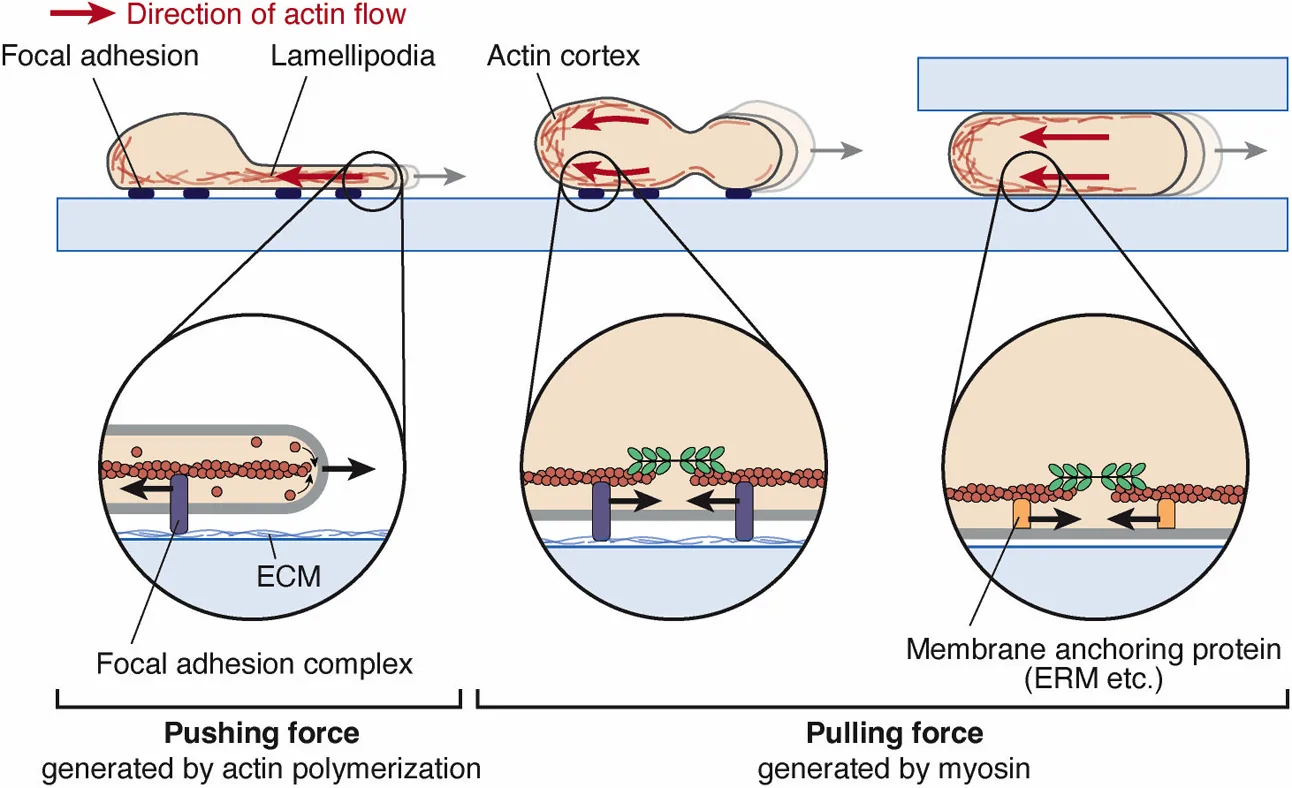
Classification of cell migration modes. Cell migration can be broadly classified into membrane protrusion driven by actin polymerization (left) and migration driven by actomyosin contraction (center and right). The latter can be further divided according to the presence (center) or absence (right) of focal adhesions. The enlarged views (bottom) illustrate the key minimal molecular elements underlying force generation. Black arrows indicate the forces exerted on the plasma membrane or membrane-associated proteins, generated either by actin polymerization (left) or actomyosin contractility (center and right).

**Figure 6:**
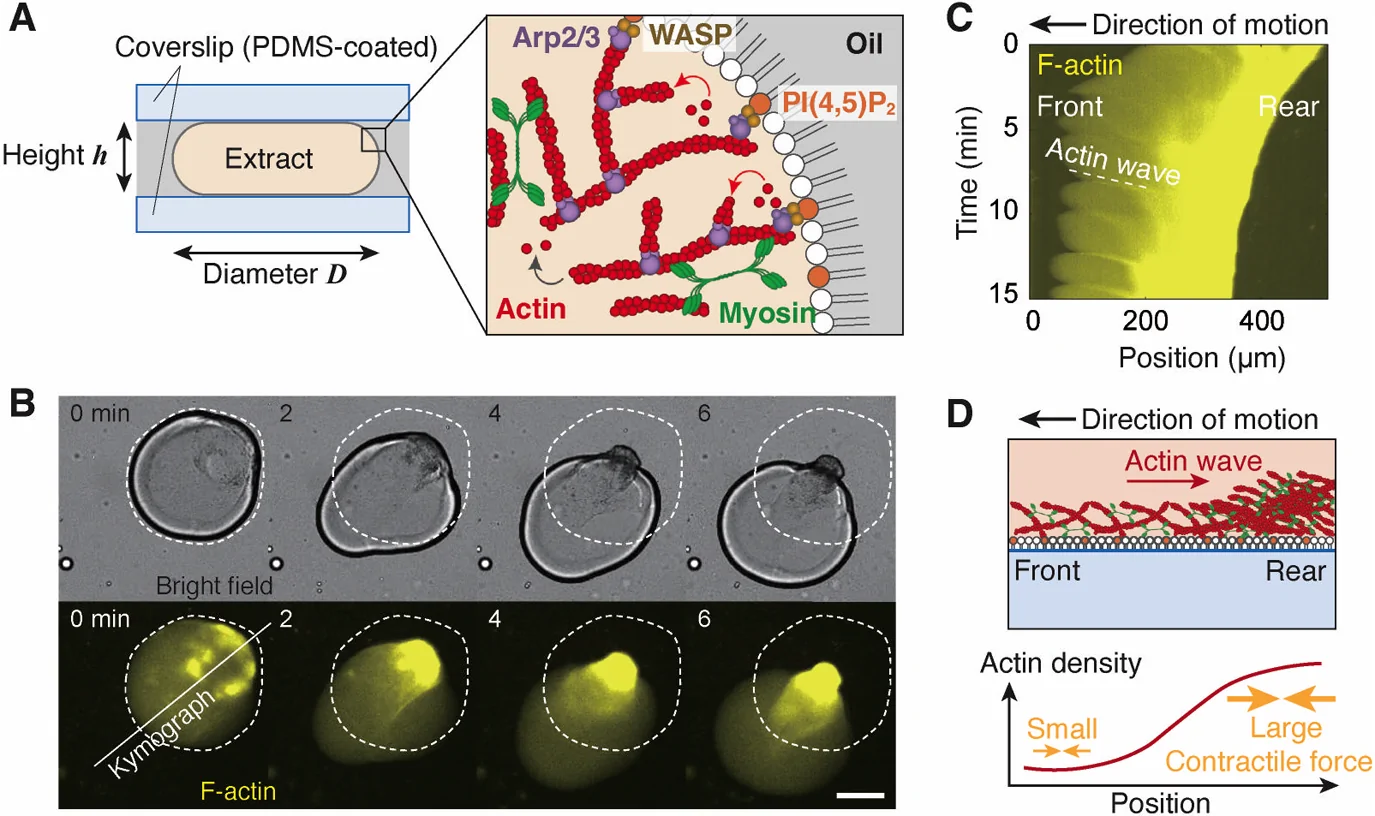
Reconstitution of actomyosin-driven cell migration. (A) Schematic illustration of the experimental system. PI(4,5)P_2_ was incorporated into the lipid membrane. (B) Time-lapse images of the droplet and (C) the corresponding kymograph of F-actin. (D) Mechanism by which an actin density gradient generates a gradient in contractile force, thereby establishing and maintaining front–rear polarity. Scale bar, 100 μm.

**Figure 7:**
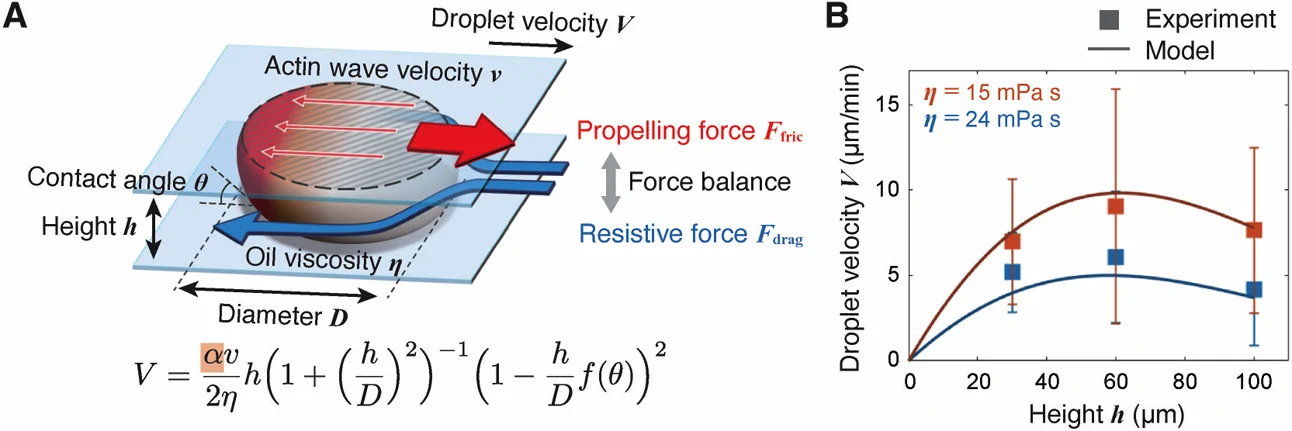
Dependence of migration velocity on confinement conditions. (A) Schematic illustration of the force balance and the equation describing droplet migration velocity derived from the force-balance model. (B) Comparison between experimental data and the theoretical model. Experimental data represent the mean migration velocity of droplets with diameters of 100–200 μm. Error bars indicate the standard deviation.
